# Genetic polymorphisms of the *CDC27* gene are associated with susceptibility and outcomes of non-syndromic congenital heart disease: a bi-ethnic case–control study in Chinese populations

**DOI:** 10.3389/fgene.2026.1701812

**Published:** 2026-02-12

**Authors:** Teng Yuan, Feng Zhu, Aikebai Aisan, Tunike Maheshati, Yunxia Li, Ren Tian, You Chen

**Affiliations:** 1 Department of Cardiology, First Affiliated Hospital of Xinjiang Medical University, Urumqi, China; 2 Department of Cardiovascular, Changshou people’s Hospital, Chongqing, China

**Keywords:** congenital heart disease, cell division cycle 27, single nucleotide polymorphism, pulmonary artery pressure, prognosis

## Abstract

**Background:**

Cell division cycle 27 (*CDC27*) gene expression is closely associated with the cell cycle and has been implicated in the pathogenesis of congenital heart disease (CHD) in animal models. This study focuses on investigating whether single-nucleotide polymorphisms (SNPs) in the *CDC27* gene are associated with CHD and the cardiac remodeling process in the population of Xinjiang, China.

**Methods:**

This study conducted a case–control study including 689 controls and 575 patients with CHD. SNPs of the *CDC27* gene were genotyped using an improved multiple ligase detection reaction.

**Results:**

Our study found that the *CDC27* rs11570579 polymorphism was significantly associated with CHD susceptibility in the Uyghur population (additive model: aOR = 0.66, *p* = 0.029; dominant model: aOR = 0.80, *p* = 0.038), but not in the Han population. The rs11570488 GA genotype was associated with higher pulmonary artery systolic pressure (PASP), more severe cardiac remodeling, and increased long-term mortality in both ethnic groups (all *p* < 0.001), with the mortality difference being significant only in patients with pulmonary hypertension. Haplotype analysis identified ethnic-specific haplotypes associated with CHD susceptibility and elevated PASP.

**Conclusion:**

The *CDC27* rs11570579 polymorphism is associated with susceptibility to CHD in the Uyghur population of Xinjiang. The rs11570488 polymorphism is associated with PASP, cardiac remodeling, and long-term mortality in patients with CHD.

## Introduction

Congenital heart disease (CHD) comprises a group of congenital malformations characterized by structural or functional abnormalities of the heart and great vessels that arise during human embryonic development. As the most common birth defect and a leading cause of non-infectious death in infants, the incidence of CHD in Asia is approximately 12.4 per 1000 live births (GBD 2017 Congenital Heart Disease Collaborators, 2020). Although significant advancements in surgical and interventional therapies in recent years have markedly reduced the mortality rate of CHD, patients may still face long-term health issues after successful correction of cardiac structural anomalies. These issues include cardiovascular complications such as arrhythmias, heart failure, and sudden cardiac death and neurodevelopmental disorders (Bouma and Mulder, 2017). Current research indicates that the pathogenesis of CHD involves the complex interplay of multiple factors, including oligogenic inheritance, epigenetic regulation, and environmental factors (Zaidi et al., 2013; Pierpont et al., 2018). Notably, polymorphisms in key transcription factors that regulate cardiac development, such as NKX2.5, TBX5, and GATA4, have been identified as closely associated with the occurrence of CHD. These findings provide important clues for a deeper understanding of the molecular mechanisms underlying CHD (Gonzá et al., 2020; Behiry et al., 2019; Fan et al., 2021).

Cell division cycle 27 gene (*CDC27* gene) encodes a core component of the anaphase-promoting complex/cyclosome (APC/C), a ubiquitin E3 ligase that primarily regulates cell cycle transitions during mitosis (Kazemi-Sefat et al., 2021). Previous studies have indicated that *CDC27* may function as either a tumor suppressor or an oncogene in various cancers, with APC/C activation implicated in its pathogenesis (Pawar et al., 2010; Qiu et al., 2017). Phosphorylation of *CDC27* is critical for APC/C activation (Huang et al., 2007). Existing evidence suggests that *CDC27* mutations may contribute to the pathogenesis of autism spectrum disorders by impairing APC/C complex function (Singh et al., 2012). The APC/C complex plays a key regulatory role in myoblast fusion and mitotic progression in myoblasts, cardiomyocytes, and pericardial cells (Drechsler et al., 2018). Recent studies using zebrafish models have further demonstrated that knockout of the *CDC27* gene leads to severe pericardial edema (Song et al., 2024). Although these findings underscore the essential role of *CDC27* in cardiac development, the association between its single-nucleotide polymorphisms (SNPs) and related phenotypic outcomes has not been systematically investigated. Therefore, the objective and primary focus of this study are to evaluate the association between *CDC27* genetic polymorphisms and CHD in the Han and Uyghur populations of Xinjiang, China.

## Materials and methods

### Study subjects

**FIGURE 1:**
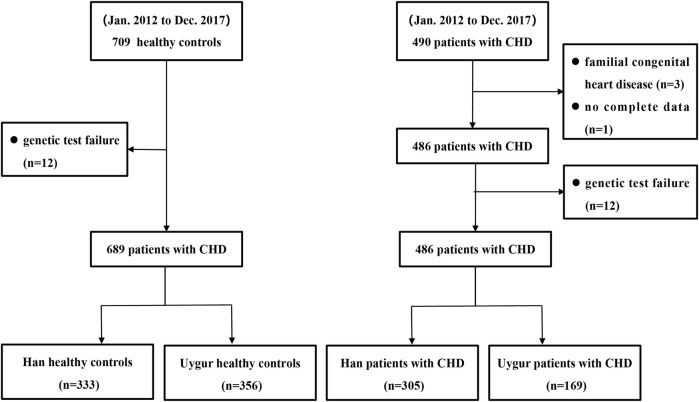
Patient enrollment flow chart.

From January 2012 to December 2017, a total of 575 patients with non-syndromic CHD were enrolled from the First Affiliated Hospital of Xinjiang Medical University, and the annual enrollment was as follows: 99 in 2012, 86 in 2013, 95 in 2014, 95 in 2015, 93 in 2016, and 107 in 2017. The diagnosis and classification of non-syndromic CHD were confirmed by echocardiography and surgical intervention. In this study, non-syndromic CHD included ventricular septal defect (VSD), atrial septal defect (ASD), atrioventricular septal defect (AVSD), patent ductus arteriosus (PDA), tetralogy of Fallot (ToF), and complete transposition of the great arteries (TGA). Patients with other organ malformations, known chromosomal abnormalities, or familial congenital heart disease were excluded from the study. During the same period, 688 healthy controls were selected from our hospital to serve as the control group. These control subjects had no diagnosed genetic disorders. To minimize residual confounding factors due to genetic and cultural differences across ethnic groups, all cases and controls were required to be of Han or Uyghur ethnicity. Additionally, both case and control subjects were required to meet the following inclusion criteria: (1) provision of blood samples; (2) completion of follow-up assessments; and (3) detectable gene sequences. The patient enrollment flowchart is presented in Figure 1.

This study was ethically conducted in accordance with the Declaration of Helsinki and was approved by the Ethics Committee of the First Affiliated Hospital of Xinjiang Medical University (approval no. 20180222-102). All participants provided written informed consent after being fully informed about the study purpose, data collection procedures, DNA analysis protocols, and telephone interview arrangements. Follow-up assessments were performed by trained medical professionals through either telephone interviews or clinical outpatient visits, with all-cause mortality serving as the primary endpoint.

### Information collection

The following clinical data were collected by two trained professionals through the electronic medical record system: age, sex, weight, ethnicity, systolic blood pressure (SBP), and diastolic blood pressure (DBP). Body weight was measured using standardized methods. Blood pressure levels were calculated as the average of two measurements taken 30 s apart. Within 6 h of hospital admission, transthoracic echocardiography was performed by an experienced cardiac sonographer using a Philips iE33 color Doppler echocardiography system (Philips Medical Systems, Eindhoven, Netherlands). Pulmonary artery systolic pressure (PASP) was measured via the tricuspid regurgitation pressure gradient method, in accordance with current clinical guidelines (Mukherjee et al., 2025). The diagnostic criterion for pulmonary hypertension is a PASP exceeding 35 mmHg. In terms of severity classification: a PASP of 35–49 mmHg indicates mild pulmonary hypertension, 50–69 mmHg represents moderate pulmonary hypertension, and values above 70 mmHg are classified as severe pulmonary hypertension. The right atrial mediolateral dimension (RA) was measured at end-systole in the apical four-chamber view as the distance from the mid-interatrial septum to the lateral wall. The right ventricular anteroposterior dimension (RV) was measured at end-diastole in the parasternal left ventricular long-axis view. The left atrial anteroposterior dimension (LA) was measured at end-systole in the parasternal long-axis view, obtained by drawing a perpendicular line from the posterior wall of the distal aorta to the posterior wall of the left atrium (Mitchell et al., 2019).

Peripheral venous blood samples were collected from all participants and stored in blood collection tubes containing ethylenediaminetetraacetic acid (EDTA). Genomic DNA was extracted from each blood sample using a Whole Blood Genome Extraction Kit (Beijing Bioteke Corporation, Beijing, China) and stored at −80 °C.

### SNP selection and genotyping

The *CDC27* gene was selected as a candidate gene for this study. Tag SNPs were selected from the Chinese Asian database of the 1000 Genomes Project (http://www.1000genomes.org/). We used Haploview 4.2 (https://www.broadinstitute.org/haploview/haploview) to identify tag SNPs with a pairwise *r* ^2^ > 0.8 and a minor allele frequency >1%. The following genetic loci were considered candidate loci in our study: rs11570488, rs11570579, rs1713494, rs221603, rs67861319, rs858678, and rs865750 of the *CDC27* gene (Table 1). The seven SNPs in the *CDC27* gene were genotyped using improved multiple ligase detection reaction (iMLDR) genotyping assays. To ensure quality control, genotyping was conducted in a blinded manner, without knowledge of the patients’ clinical characteristics. A randomly selected subset of samples (10%) underwent repeated genotyping for quality assessment.

**TABLE 1:** Baseline clinical characteristics of the two groups.

Variable	Han			Uyghur		
	Case	Control	*p*-value	Case	Control	*p*-value
	(N = 304)	(N = 333)		(N = 271)	(N = 355)	
Age, years	40.14 ± 17.85	54.01 ± 7.76	**<0.001**	40.76 ± 14.11	51.55 ± 10.21	**<0.001**
Gender, N (%)	​	​	**<0.001** ^a^	​	​	**<0.001** ^a^
Male	100 (33.0)	163 (48.9)	​	81 (29.9)	185 (52.1)	​
Female	203 (67.0)	170 (51.1)	​	190 (70.1)	170 (47.9)	​
SBP, mmHg	114.33 ± 14.66	116.16 ± 13.05	0.095	115.67 ± 14.72	117.40 ± 12.71	0.117
DBP, mmHg	71.27 ± 8.36	72.88 ± 8.46	**0.016**	70.85 ± 8.49	73.45 ± 8.19	**<0.001**
body weight	59.78 ± 15.75	68.92 ± 7.99	**<0.001**	64.45 ± 13.07	71.33 ± 8.78	**<0.001**
PASP, mmHg	32 (23, 47)	23 (21, 24)	**<0.001** ^b^	25 (22, 45)	22 (21, 24)	**<0.001** ^b^

SBP, systolic blood pressure; DBP, diastolic blood pressure; PASP, pulmonary artery systolic pressure.

^a^ *p*-values were calculated using the chi-square test. Other variables were calculated using the t-test.

^b^ *p*-values were calculated using the Mann–Whitney U test.

Bold indicates statistical significance.

### Follow-up

The primary endpoint of this study was all-cause mortality. All CHD patients were required to undergo structured telephone interviews to confirm all-cause mortality. If direct contact with a patient was not possible, their family members were interviewed. All hospitalization data were cross-referenced with hospital medical records. Deaths attributable to direct cardiac causes (e.g., low cardiac output failure and fatal arrhythmias), unwitnessed deaths, deaths of unknown cause, and procedure-related deaths (including deaths associated with concomitant therapies) were all classified as all-cause mortality.

### Statistical analyses

Haploview 4.2 software was employed for linkage disequilibrium (LD) testing and haplotype analysis. Additional statistical analyses were conducted using R software (version 4.4.1). The Kolmogorov–Smirnov test was used to assess the normality of continuous variables. Normally distributed continuous variables were expressed as the mean ± SD and compared using independent samples t-tests. Non-normally distributed continuous variables were presented as the median (interquartile range, IQR) and analyzed using the Mann–Whitney U test. Categorical variables were described as frequencies (percentages), with between-group comparisons performed using chi-square tests. The chi-square test was also used to evaluate the Hardy–Weinberg equilibrium (HWE) for *CDC27* gene SNPs in the control group. Univariate logistic regression was performed to calculate odds ratios (ORs) with 95% confidence intervals (CIs), assessing associations between *CDC27* gene polymorphisms and CHD risk. Multivariable logistic regression was subsequently conducted to compute adjusted ORs (aORs), controlling for statistically significant covariates between cases and controls, thereby evaluating independent associations between *CDC27* SNPs and CHD susceptibility. Genetic associations were analyzed under three genetic models: dominant, recessive, and additive. Kaplan–Meier (K-M) curve analysis was employed to assess the association between *CDC27* SNPs and CHD prognosis. LD analysis was used to determine potential correlations between SNPs. Associations between haplotypes and the risk of CHD were estimated by multivariable logistic regression. All statistical tests were two-sided, with *p* < 0.05 considered statistically significant.

## Results

### Clinical characteristics

This study evaluated two ethnic groups within the Chinese population: Han and Uyghur. Among Han Chinese participants with CHD, 219 cases were identified with ASD, 46 cases with VSD, and 39 cases with PDA. In the Uyghur participants with CHD, the corresponding frequencies were 213 cases with ASD, 35 cases with VSD, and 23 cases with PDA. The clinical characteristics of the two study populations are presented in Table 1. Significant differences were observed between the CHD and control groups in terms of age, sex, DBP, and body weight among both Han and Uyghur participants (all *p* < 0.05). In the CHD group, patients exhibited lower age, DBP, and body weight than those of the control group, and the proportion of female CHD patients was higher than that in the control group.

### Association of *CDC27* polymorphisms with susceptibility, cardiac remodeling, and prognosis of CHD

The association analysis between each SNP of *CDC27* and CHD susceptibility was presented in Tables 2–4. All SNPs in both study populations were in Hardy–Weinberg equilibrium (all *p* > 0.05). After adjustment for age, sex, SBP, DBP, and body weight, the genetic polymorphism of *CDC27* at rs11570579 showed a significant association with CHD risk in the Uyghur population (additive model: aOR = 0.66, 95% CI = 0.45–0.95, *p* = 0.029, FDR-*p* = 0.038; dominant model: aOR = 0.80, 95% CI = 0.62-0.98, *p* = 0.038, FDR-*p* = 0.038). In contrast, no significant association was observed between *CDC27* genetic polymorphisms and CHD risk in the Han Chinese population.

**TABLE 2:** Genotype distribution and Hardy–Weinberg equilibrium analysis of CDC27 polymorphisms in Han and Uyghur congenital heart disease patients and controls.

Genotype	Genomic position (GRCh38.p14)	Han cases		Han controls		Uyghur cases		Uyghur controls	
		MAF	HWE *p*-value	MAF	HWE *p*-value	MAF	HWE *p*-value	MAF	HWE *p*-value
rs11570488 (G>A)	chr17:47155406	0.085	0.338	0.075	0.999	0.051	0.601	0.06	0.841
rs11570579(C>T)	chr17:47119930	0.176	0.947	0.213	0.774	0.121	0.914	0.173	0.998
rs1713494 (T>C)	chr17:47118824	0.143	0.774	0.182	0.753	0.249	0.974	0.232	0.914
rs221603(C>T)	chr17:47172271	0.344	0.938	0.411	0.999	0.448	0.451	0.448	0.901
rs67861319 (G>A)	chr17:47147591	0.446	0.934	0.426	0.426	0.345	0.776	0.365	0.52
rs858678(C>G)	chr17:47163605	0.138	0.553	0.105	0.69	0.175	0.94	0.153	0.968
rs865750 (A>C)	chr17:47149976	0.338	0.734	0.309	0.332	0.289	0.931	0.307	0.887

MAF, minor allele frequency; HWE, Hardy–Weinberg equilibrium.

**TABLE 3:** Genetic polymorphisms of the *CDC27* gene and the risk of CHD in the Han group.

Genotype	Cases N (%)	Controls N (%)	Adjusted OR (95% CI)^a^	*p*-value^a^	Models	Adjusted OR (95% CI)^a^	*p*-value^a^
rs11570488 (G>A)							
GG	252 (82.9)	285 (85.6)	Ref	​	Add	1.29 (0.80–2.09)	0.291
GA	52 (17.1)	46 (13.8)	1.48 (0.89–2.45)	0.129	Rec	NA	NA
AA	0 (0)	2 (0.6)	NA	NA	Dom	1.18 (0.92–1.52)	0.188
rs11570579(C>T)							
CC	205 (67.4)	203 (61.0)	Ref	​	Add	0.79 (0.56–1.12)	0.181
TC	91 (29.9)	118 (35.4)	0.82 (0.55–1.22)	0.334	Rec	0.75 (0.42–1.32)	0.324
TT	8 (2.6)	12 (3.6)	0.52 (0.16–1.66)	0.274	Dom	0.89 (0.73–1.08)	0.243
rs1713494(T>C)							
TT	221 (72.7)	226 (67.9)	Ref	​	Add	0.79 (0.58–1.13)	0.199
CT	79 (26.0)	93 (27.9)	0.92 (0.61–1.40)	0.726	Rec	0.55 (0.28–1.07)	0.08
CC	4 (1.3)	14 (4.2)	0.3 (0.08–1.14)	0.076	Dom	0.91 (0.75–1.12)	0.412
rs221603(C>T)							
CC	133 (43.8)	115 (34.5)	Ref	​	Add	0.78 (0.60–1.03)	0.081
CT	133 (43.8)	162 (48.6)	0.74 (0.49–1.10)	0.141	Rec	0.87 (0.67–1.13)	0.306
TT	38 (12.5)	56 (16.8)	0.64 (0.37–1.14)	0.163	Dom	0.78 (0.60–1.03)	0.081
rs67861319(G>A)							
GG	91 (29.9)	118 (35.4)	Ref	​	Add	1 (0.77–1.29)	0.999
GA	155 (51.0)	146 (43.8)	1.11 (0.73–1.69)	0.602	Rec	0.95 (0.76–1.19)	0.687
AA	58 (19.1)	69 (20.8)	0.97 (0.57–1.63)	0.916	Dom	1.03 (0.85–1.26)	0.729
rs858678(C>G)							
CC	223 (73.4)	265 (79.6)	Ref	​	Add	1.41 (0.91–2.13)	0.099
CG	78 (25.7)	66 (19.8)	1.42 (0.91–2.22)	0.114	Rec	1.27 (0.47–3.41)	0.625
GG	3 (1)	2 (0.6)	1.71 (0.25–12.80)	0.562	Dom	1.2 (0.96–1.49)	0.1
rs865750(A>C)							
AA	137 (45.1)	151 (45.3)	Ref	​	Add	1.07 (0.79–1.44)	0.639
AC	139 (45.7)	158 (47.4)	0.97 (0.66–1.42)	0.896	Rec	1.17 (0.83–1.67)	0.3559
CC	28 (9.2)	24 (7.2)	1.37 (0.66–2.83)	0.397	Dom	1.01 (0.84–1.21)	0.919

Abbreviations: OR, odds ratio; CI, confidence interval; ORs and 95% CIs were calculated using logistic regression. Add model, additive model; Rec model, recessive model; Dom model, dominant model.

^a^ Adjusted for age, gender, diastolic blood pressure, body weight, and systolic blood pressure.

**TABLE 4:** Genetic polymorphisms of the *CDC27* gene and the risk of CHD in the Uyghur group.

Genotype	Cases N (%)	Controls N (%)	Adjusted OR (95% CI)	*p*-value^a^	Models	Adjusted OR (95% CI)	*p*-value^a^
rs11570488 (G>A)							
GG	243 (89.7)	314 (88.5)	Ref	​	Add	0.84 (0.47–1.47)	0.542
GA	28 (10.3)	39 (11.0)	0.91 (0.51–1.65)	0.771	Rec	NA	NA
AA	0 (0)	2 (0.5)	NA	NA	Dom	0.93 (0.69–1.25)	0.651
rs11570579(C>T)							
CC	208 (76.8)	243 (68.5)	Ref	​	Add	**0.66 (0.45–0.95)**	**0.029**
TC	60 (22.1)	101 (28.4)	0.67 (0.43–1.02)	0.067	Rec	0.66 (0.334–1.31)	0.238
TT	3 (1.1)	11 (3.1)	0.39 (0.10–1.56)	0.188	Dom	**0.80 (0.62–0.98)**	**0.038**
rs1713494(T>C)							
TT	154 (56.8)	211 (59.4)	Ref	​	Add	1.06 (0.78–1.42)	0.699
CT	99 (36.5)	123 (34.6)	1.01 (0.69–1.49)	0.932	Rec	1.09 (0.76–1.58)	0.62
CC	18 (6.6)	21 (6.0)	1.21 (0.57–2.56)	0.615	Dom	1.02 (0.85–1.22)	0.814
rs221603(C>T)							
CC	75 (27.7)	105 (29.5)	Ref	​	Add	1.02 (0.78–1.34)	0.843
CT	149 (55.0)	182 (51.3)	1.19 (0.78–1.82)	0.406	Rec	0.95 (0.75–1.20)	0.666
TT	47 (17.3)	68 (19.2)	1.01 (0.58–1.74)	0.964	Dom	1.07 (0.87–1.31)	0.506
rs67861319(G>A)							
GG	120 (44.3)	136 (38.3)	Ref	​	Add	0.88 (0.67–1.15)	0.369
GA	115 (42.4)	179 (50.4)	0.74 (0.51–1.09)	0.138	Rec	1.02 (0.78–1.34)	0.856
AA	36 (13.3)	40 (11.3)	0.91 (0.50–1.61)	0.727	Dom	0.88 (0.73–1.05)	0.177
rs858678(C>G)							
CC	183 (67.5)	255 (71.8)	Ref	​	Add	1.19 (0.84–1.69)	0.308
CG	81 (29.9)	91 (25.6)	1.22 (0.81–1.85)	0.326	Rec	1.11 (0.63–1.91)	0.721
GG	7 (2.6)	9 (2.5)	1.29 (0.43–3.90)	0.644	Dom	1.11 (0.91–1.35)	0.298
rs865750(A>C)							
AA	135 (49.8)	173 (48.8)	Ref	​	Add	0.93 (0.71–1.24)	0.656
AC	115 (42.4)	146 (41.1)	0.97 (0.66–1.42)	0.882	Rec	0.92 (0.66–1.27)	0.62
CC	21 (7.8)	36 (10.1)	0.83 (0.42–1.63)	0.605	Dom	0.97 (0.81–1.16)	0.768

Abbreviations: OR, odds ratio; CI, confidence interval; ORs and 95% CIs were calculated using logistic regression. Add model, additive model; Rec model, recessive model; Dom model, dominant model.

^a^ Adjusted for age, gender, diastolic blood pressure, body weight, and systolic blood pressure.

Bold indicates statistical significance.

**FIGURE 2:**
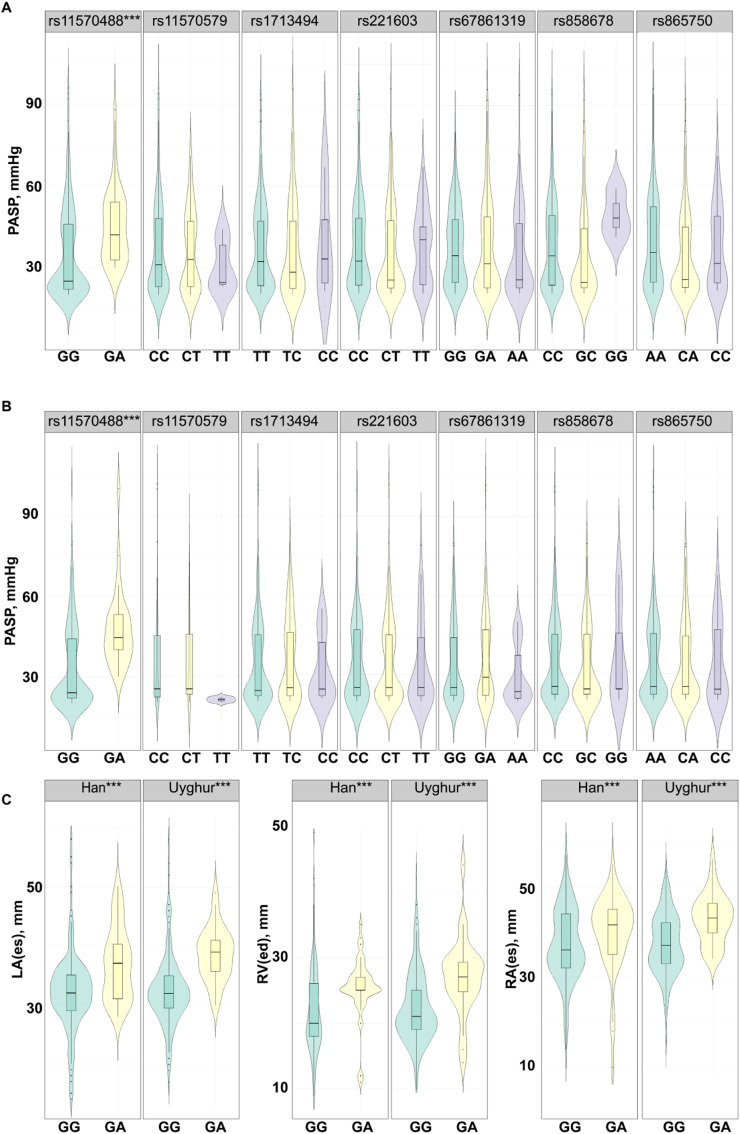
Differences in PASP and cardiac remodeling among different genotypes of candidate SNPs of the *CDC27* gene **(A,B)**. Violin plot showing the distribution of PASP among different genotypes of the candidate SNP in Han **(A)** and Uyghur **(B)** subjects with CHD. ^***^ *p* < 0.001. The width represents data density, and the inner box plot displays the interquartile range and median **(C)**. Association between the rs11570488 polymorphism and cardiac remodeling. ^***^ *p* < 0.001. PASP, pulmonary artery systolic pressure; LA, left atrial diameter; RV, right ventricular end-diastolic dimension, RA, right atrial diameter.

We further analyzed the association between *CDC27* gene polymorphisms and pulmonary artery pressure in patients with CHD, as illustrated in Figures 2A,B. In the Han population, the median PASP of subjects with the *CDC27* rs11570488 GA genotype was 42 (32.25-54) mmHg, compared to 25 (22-46) mmHg in those with the GG genotype. Similarly, in the Uyghur population, the median PASP was 44.5 (40–53) mmHg for GA genotype carriers versus 24 (22–44) mmHg for GG genotype carriers. Among both Han and Uyghur patients with CHD, subjects carrying the GA genotype exhibited significantly higher PASP levels than those with the GG genotype (all *p* < 0.001). Additionally, the *CDC27* rs11570488 polymorphism was significantly associated with cardiac remodeling in patients with CHD (Figure 2C). In both ethnic groups, GA genotype carriers demonstrated significantly larger LA diameter, RV, and RA diameter than GG genotype carriers (all *p* < 0.001). These findings suggest that the rs11570488 GG genotype may be associated with higher PASP levels and more pronounced cardiac remodeling, with this trend being consistent across different ethnicities.

**FIGURE 3:**
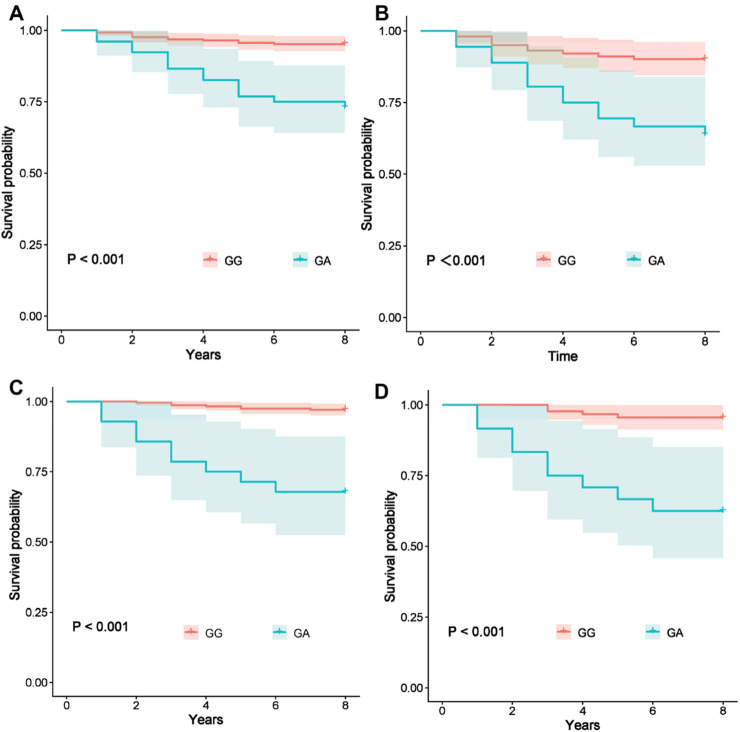
Association between the *CDC27* rs11570488 polymorphism and long-term mortality risk in patients with CHD **(A–D)** Kaplan–Meier curves for long-term mortality across different genotypes of the *CDC27* rs11570488 polymorphism. **(A)** All Han CHD patients, **(B)** Han CHD patients with pulmonary hypertension, **(C)** all Uyghur CHD patients, and **(D)** Uyghur CHD patients with pulmonary hypertension.

Previous studies have confirmed that pulmonary hypertension significantly impacts the prognosis of CHD. We further investigated the association between the *CDC27* gene rs11570488 SNP and long-term mortality risk in CHD patients. This study completed a follow-up of 8–13 years for all enrolled patients. To ensure data consistency, the survival analysis was based solely on patients who had all completed the 8-year follow-up. The results showed that there were 42 deaths (7.3%) in the overall population during the 8-year follow-up period. Among the Han patients, 26 deaths (8.5%) occurred, with 14 (26.9%) and 12 (4.7%) in those with the GA and GG genotypes, respectively. Among the Uyghur patients, 16 deaths (5.9%) occurred, with 9 (32.1%) and 7 (2.8%) in those with the GA and GG genotypes, respectively. Kaplan–Meier survival analysis (Figure 3A) revealed that CHD patients carrying the GA genotype had significantly higher long-term mortality than those with the GG genotype in both Han and Uyghur populations (*p* < 0.001). Among Han patients with CHD and concomitant pulmonary hypertension, the mortality rate was 13 (36.1%) in those with the GA genotype and 1 (9.9%) in those with the GG genotype. In contrast, among Han patients without pulmonary hypertension, the mortality rate was 1 (6.2%) in the GA genotype group and 2 (1.3%) in the GG genotype group. Among Uyghur patients with CHD and pulmonary hypertension, mortality was observed in 9 (37.5%) with the GA genotype and 4 (4.4%) with the GG genotype. For Uyghur patients without pulmonary hypertension, no deaths (0%) occurred in the GA genotype group, whereas 3 (1.9%) deaths occurred in the GG genotype group. Notably, in patients with concomitant pulmonary hypertension, those with the GA genotype exhibited a significantly higher mortality risk compared to GG genotype carriers (*p* < 0.001) (Figures 3B,D). These findings suggest that the impact of the *CDC27* rs11570488 polymorphism on CHD prognosis may be influenced by the presence of pulmonary hypertension.

### Association of *CDC27* haplotype with susceptibility and cardiac remodeling in CHD

**FIGURE 4:**
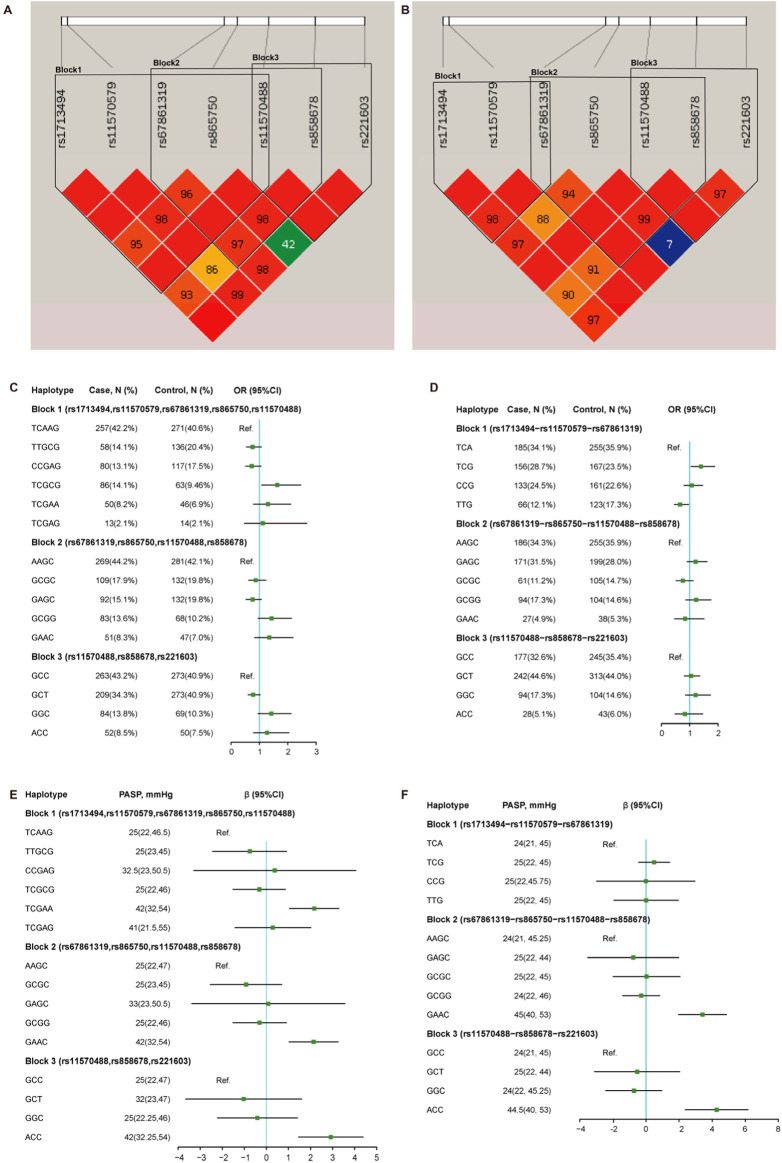
Linkage disequilibrium and haplotype analysis of candidate SNPs in the *CDC27* gene in relation to CHD. **(A,B)** LD plots constructed from candidate SNPs in the Han **(A)** and Uyghur **(B)** populations. **(C,D)** After adjusting for age, gender, diastolic blood pressure, body weight, and systolic blood pressure, association analysis between different haplotypes and CHD susceptibility in the Han **(C)** and Uyghur **(D)** populations. **(E,F)** After adjusting for age, gender, diastolic blood pressure, body weight, and systolic blood pressure, association between different haplotypes and PASP in patients with CHD from the Han **(E)** and Uyghur **(F)** populations. PASP, pulmonary artery systolic pressure.

As illustrated in Figures 4A,B, this study identified three potential linkage disequilibrium (LD) blocks composed of target SNPs in both Han and Uyghur populations. The associations between *CDC27* gene haplotypes (comprising seven SNPs) and CHD risk are presented in Figures 4C,D. Among Uyghur participants, after adjusting for age, sex, systolic blood pressure, diastolic blood pressure, and body weight, the T-C-G haplotype in Block 1 (involving rs1713494, rs11570579, and rs67861319) was significantly associated with CHD susceptibility (aOR = 1.40, 95% CI = 1.05–1.88, *p* = 0.023). Conversely, the T-T-G haplotype exhibited a protective effect (aOR = 0.66, 95% CI = 0.46–0.96, *p* = 0.032). Among Han participants, the T-C-G-C-G haplotype in Block 1 (comprising rs1713494, rs11570579, rs67861319, rs865750, and rs11570488) was significantly associated with increased CHD risk (aOR = 1.63, 95% CI = 1.08–2.46, *p* = 0.019).

Further analysis was conducted to investigate the association between haplotypes and pulmonary artery pressure in patients with CHD. As illustrated in Figures 4E,F, after adjusting for age, sex, systolic blood pressure, diastolic blood pressure, and body weight, the G-A-A-C haplotype in Block 2 (involving rs67861319, rs865750, rs11570488, and rs858678) and the A-C-C haplotype in Block 3 (involving rs11570488, rs858678, and rs221603) were significantly associated with elevated PASP in both Han and Uyghur populations. Additionally, the T-C-G-A-A haplotype in Block 1 (involving rs1713494, rs11570579, rs67861319, rs865750, and rs11570488) demonstrated a significant correlation with higher PASP values specifically in Han subjects, while this association was not observed in the Uyghur population.

### Subgroup analysis

The results of subgroup analyses based on sex and CHD type are presented in Tables 5, 6. A significant association between the CDC27 rs11570579 and CHD risk was also observed in the Uyghur male subgroup and the Uyghur ASD subgroup under both the additive and dominant models (all *p* < 0.05). Furthermore, across multiple subgroups—including Han male individuals, Han female individuals, Uyghur male individuals, Uyghur female individuals, Han ASD, and Uyghur ASD—it was consistently found that patients carrying the GA genotype of the CDC27 rs11570488 exhibited more severe ventricular remodeling and higher PASP compared to those carrying the GG genotype.

**TABLE 5:** Subgroup analysis of the association between the CDC27 gene rs11570579 polymorphism and CHD risk.

Genotype	Cases N (%)	Controls N (%)	Adjusted OR (95% CI)	*p*-value^a^	Model	Adjusted OR (95% CI)	*p*-value^a^
Han male patients with CHD vs. Han male controls							
CC	67 (66.4)	96 (58.8)	Ref	​	Add	0.74 (0.44, 1.25)	0.262
TC	32 (31.6)	60 (36.8)	0.88 (0.48,1.61)	0.684	Rec	0.48 (0.18,1.30)	0.152
TT	2 (2.0)	7 (4.4)	0.22 (0.03,1.63)	0.141	Dom	0.89 (0.66,1.20)	0.459
Han female patients with CHD vs. Han female controls							
CC	107 (62.9)	138 (67.1)	Ref	​	Add	0.80 (0.50,1.27)	0.345
TC	58 (34.1)	59 (20.0)	0.76 (0.44,1.31)	0.325	Rec	0.92 (0.43,1.98)	0.849
TT	5 (3.0)	6 (2.9)	0.79 (0.17,3.63)	0.762	Dom	0.87 (0.67,1.13)	0.316
Uyghur male patients with CHD vs. Uyghur male controls							
CC	124 (67.0)	66 (88.5)	Ref	​	Add	**0.48 (0.25,0.89)**	**0.02**
TC	53 (28.6)	15 (18.5)	0.62 (0.30,1.26)	0.19	Rec	NA	NA
TT	8 (4.4)	0 (0)	NA	NA	Dom	**0.68 (0.49,0.93)**	**0.01**
Uyghur female patients with CHD vs. Uyghur female controls							
CC	119 (70.0)	142 (74.8)	Ref	​	Add	0.86 (0.52,1.41)	0.548
TC	48 (28.3)	45 (23.6)	0.77 (0.44,1.35)	0.371	Rec	1.19 (0.49,2.87)	0.698
TT	3 (1.7)	3 (1.6)	1.33 (0.22,7.80)	0.752	Dom	0.89 (0.68,1.17)	0.443
Han individuals with ASD vs. Han controls							
CC	145 (66.2)	203 (61.0)	Ref	​	Add	0.79 (0.54,1.14)	0.214
TC	69 (31.5)	118 (35.4)	0.80 (0.52,1.23)	0.322	Rec	0.79 (0.42,1.45)	0.448
TT	5 (2.3)	12 (3.6)	0.57 (0.16,1.97)	0.383	Dom	0.88 (0.71,1.09)	0.253
Uyghur individuals with ASD vs. Uyghur controls							
CC	165 (77.4)	243 (68.5)	Ref	​	Add	**0.65 (0.45,0.92)**	**0.017**
TC	45 (21.2)	101 (28.5)	0.71 (0.45,1.11)	0.14	Rec	0.73 (0.37,1.46)	0.38
TT	3 (1.4)	11 (3.0)	0.49 (0.12,1.96)	0.318	Dom	**0.79 (0.65,0.96)**	**0.021**

Abbreviations: OR, odds ratio; CI, confidence interval; ORs and 95% CIs were calculated using logistic regression. Add model, additive model; Rec model, recessive model; Dom model, dominant model; CHD, congenital heart disease; ASD, atrial septal defect.

^a^ Adjusted for age, diastolic blood pressure, body weight, and systolic blood pressure.

Bold indicates statistical significance.

**TABLE 6:** Subgroup analysis of the CDC27 rs11570488 polymorphism with cardiac remodeling and prognosis in CHD.

Genotype	PASP, mmHg	LA (es), mm	RA (es), mm	RV (ed), mm
Han male patients with CHD				
GG	23 (20,26.5)	32.61 ± 8.13	36.61 ± 8.69	21.89 ± 7.20
GA	25 (22,29)	37.2 ± 5.38	40.86 ± 8.21	25.2 ± 4.58
*p*-value	**0.001** ^a^	**0.01**	0.082	**0.027**
Han female patients with CHD				
GG	23 (21,27.25)	31.68 ± 6.29	37.46 ± 7.59	21.75 ± 5.71
GA	25 (22,30)	36.81 ± 6.38	39.37 ± 8.48	25.48 ± 4.52
*p*-value	**<0.001** ^a^	**<0.001**	0.177	**<0.001**
Uyghur male patients with CHD				
GG	23 (20,25)	34.05 ± 7.24	36.51 ± 4.43	22.27 ± 5.39
GA	23 (20,26)	37.8 ± 2.16	42.8 ± 7.19	29 ± 4.49
*p*-value	**0.008** ^a^	**0.013**	**0.039**	**0.008**
Uyghur female patients with CHD				
GG	23 (20,25)	31.7 ± 5.07	37.68 ± 6.42	22.25 ± 5.23
GA	27 (25,31)	38.78 ± 4.87	42.91 ± 5.16	26.65 ± 6.13
*p*-value	**<0.001** ^a^	**<0.001**	**<0.001**	**<0.001**
Han individuals with ASD				
GG	24 (21,27)	32.28 ± 6.62	39.2 ± 7.73	23.5 ± 6.14
GA	25 (22,29)	35.94 ± 5.53	41.48 ± 7.62	26.45 ± 3.11
*p*-value	**<0.001** ^a^	**0.001**	0.111	**<0.001**
Uyghur individuals with ASD				
GG	23 (21,25)	32.25 ± 5.55	38.47 ± 5.97	22.93 ± 5.34
GA	28 (25,31)	38.23 ± 5.29	43.05 ± 4.85	28.35 ± 6.26
*p*-value	**<0.001** ^a^	**<0.001**	**0.002**	**<0.001**

Abbreviations: CHD, congenital heart disease; ASD, atrial septal defect; K-M, Kaplan–Meier.

^a^ *p*-values were calculated using a Mann–Whitney U test. Other variables were calculated using t-test.

Bold indicates statistical significance.

## Discussion

In this study, we investigated the association between common SNPs in the human *CDC27* gene and susceptibility to CHD as well as PASP in Han and Uyghur populations. This is the first study to explore the relationship between common variants in the *CDC27* gene and susceptibility to sporadic non-syndromic CHD. The results revealed that the rs11570579 polymorphism was significantly associated with CHD susceptibility in the Uyghur population, while no such association was observed in the Han population. Furthermore, the rs11570488 polymorphism was found to be correlated with PASP levels in CHD patients from both ethnic groups, suggesting that this locus may play a potential role in the regulation of pulmonary artery pressure across different populations.

The *CDC27* gene comprises 33 specific exons and can generate multiple transcripts through alternative splicing, among which 13 spliced mRNAs are predicted to encode functional proteins. The major functional isoforms of *CDC27* consist of 830 and 824 amino acids, respectively, and are encoded by 19 exons. These proteins contain two tetratricopeptide repeat (TPR) domains, with five TPR motifs in the N-terminal and nine in the C-terminal domain. As a core component of the APC/C, *CDC27* plays a critical regulatory role in cell cycle transitions during cell division (Kazemi-Sefat et al., 2021). Previous studies have indicated that *CDC27* may function either as a tumor suppressor or as an oncogene in different types of cancer, depending on the activation status of the APC/C (Pawar et al., 2010; Qiu et al., 2017). It has also been implicated in the pathogenesis of various tumors. Somatic mutations in *CDC27* have been identified in a wide range of human solid malignancies, including non-small cell lung cancer, gastric cancer, colorectal cancer, thyroid cancer, and breast cancer (Wu et al., 2020; Matejcic et al., 2025; Henarejos-Castillo et al., 2024; Zheng et al., 2021; Vellichirammal et al., 2023). In the Chinese non-small cell lung cancer population, the mutation frequency of the *CDC27* gene is as high as 15.2% (Zheng et al., 2021). In malignant tumors, *CDC27* mutations are generally associated with poor prognosis, including higher risks of metastasis and lower survival rates (Qiu et al., 2017). Their biological mechanism may be linked to dysregulation of the APC/C complex, leading to cell cycle abnormalities and genomic instability. Mutations in the coding region may indicate an unfavorable prognosis, which could be attributed to the high conservation of the *CDC27* gene—any amino acid change in this region is likely to severely impair its function, resulting in significant biological consequences. Therefore, we speculate that variants in the coding region of *CDC27* may not be common pathogenic factors in sporadic non-syndromic CHD. However, animal studies have shown that *CDC27*-knockout zebrafish develop severe pericardial edema 3 days post fertilization, suggesting that this gene may play a critical role in cardiac development (Song et al., 2024). Based on this finding, the present study aimed to investigate whether common population genetic variants in the *CDC27* gene are associated with susceptibility to non-syndromic sporadic CHD in the Xinjiang region of China. After multivariate adjustment, our findings revealed a significant association between the rs11570579 polymorphism in the *CDC27* gene and CHD susceptibility. rs11570579 is located in the 3′untranslated region (3′UTR) of the *CDC27* gene. Although it does not encode proteins, the 3′UTR is a crucial regulator of gene expression, primarily by controlling mRNA stability, localization, and translation efficiency (Griesemer et al., 2021). Dysregulation of the 3′UTR represents an important disease mechanism. Specifically, genetic variations in this region can create or disrupt binding sites for microRNAs and RNA-binding proteins, leading to aberrant translational inhibition or reduced mRNA stability, which ultimately alters protein expression levels. Additionally, mutations may impair the polyadenylation signal, resulting in abnormal mRNA processing. Such mechanisms have been associated with various disorders, including systemic lupus erythematosus, multiple sclerosis, and coronary artery disease (Chan et al., 2023). This observational study further demonstrates that the rs11570579 polymorphism in the *CDC27* gene is associated with the risk of non-syndromic CHD in the Uyghur population of Xinjiang, China, under both additive and dominant genetic models.

Further correlation analysis on CHD phenotypes revealed an association between the *CDC27* gene rs11570488 polymorphism and PASP in CHD patients. rs11570488 is located in intron 7 of the *CDC27* gene. In eukaryotic genomes, introns account for a large proportion of the sequence, and this region contains a considerable number of SNPs. However, compared to coding regions and gene regulatory regions, SNP located in intronic regions generally confer lower pathogenic risk (Nascimbeni et al., 2010). Although introns do not encode proteins, they serve as an indispensable “control center” for precise gene expression, and their dysfunction can lead to disease through multiple mechanisms. The most central mechanism involves disrupting mRNA splicing. Mutations in the canonical splice site sequences at intron boundaries or in fine-tuning regulatory elements within introns can compromise splicing fidelity. This leads to aberrant events such as exon skipping or intron retention, ultimately generating incorrect mRNA transcripts and producing dysfunctional proteins (Anna and Monika, 2018). Furthermore, introns often contain critical transcriptional regulatory elements, such as enhancers and silencers, along with genes for non-coding RNAs, including miRNAs. Mutations within these regions can directly interfere with transcriptional activity or disrupt broader regulatory networks (Zh et al., 2022). Therefore, genetic variations in intronic regions play a critical role in the pathogenesis of various hereditary diseases and even cancers by collectively interfering with multiple processes, including transcription, splicing, and gene expression regulation (Vaz-Drago et al., 2017; Jolma et al., 2013). This observational study showed that CHD patients carrying the GA genotype of rs11570488 had significantly higher PASP than those with the GG genotype. Additionally, patients with the GA genotype exhibited larger left atrial, right atrial, and right ventricular diameters compared with those with the GG genotype. Long-term follow-up results further indicated that CHD patients with the GA genotype had a higher risk of long-term mortality. Notably, these differences were only observed in CHD patients complicated with pulmonary hypertension, suggesting that this genotype may predispose CHD patients with pulmonary hypertension to more pronounced cardiac remodeling. However, no rare homozygous genotype was observed at the rs11570488 locus in this study. We speculate that this may be because individuals with a homozygous genotype at this locus could experience more severe pulmonary hypertension, have a shorter survival time, and may have been treated at other medical institutions or died before birth. Currently, research on the functions of the intronic and 3′UTR regions of the *CDC27* gene remains relatively limited. Future genome-wide sequencing studies in larger populations, along with in-depth functional experiments, will help elucidate its specific role in the disease mechanism.

This study has several limitations. First, all samples were collected from a single medical center, and the number of homozygous individuals for rare genotypes was limited, which may have led to sample selection bias. Second, although environmental factors during parental pregnancy may be associated with susceptibility to CHD, such covariates were not included in the analysis due to practical challenges in obtaining pregnancy-related data. Finally, the specific molecular mechanisms through which these SNPs influence the pathogenesis of CHD remain unclear and require further functional experimental validation.

## Data Availability

The original contributions presented in the study are included in the article/Supplementary Material, further inquiries can be directed to the corresponding author.
