# Supplementary material for: Genetic polymorphisms of the CDC27 gene are associated with susceptibility and outcomes of non-syndromic congenital heart disease: a bi-ethnic case–control study in Chinese populations
Source: Front Genet. 2026 Feb 12;17:1701812. doi: 10.3389/fgene.2026.1701812 (PMC12935316; doi:10.3389/fgene.2026.1701812)
Supplement: Supplementary file 1 [file Table1.docx]

*Supplement Paper*

Table S1. PCR primers sequence

| Name | Sequence (5’-3’) |
| --- | --- |
| rs11570488F | CTATTTTAGTAGTTCCCCGGCCTCA |
| rs11570488R | CAGGAACTCAGCCCAGAGGATACA |
| rs11570579F | CCCCAAGCTAAAAATTTCCAACATC |
| rs11570579R | TTCCCCACTGAGAAGCCAAAAC |
| rs1713494F | GATCCCCATTCCATGCAAAAAA |
| rs1713494R | GGCAAAGCACAAGGTGCTGTAT |
| rs221603F | TGGAGAAACGGTTTGCTTATAGCAA |
| rs221603R | GTCTGTTTAAAGGCAGGGGTGTTTC |
| rs67861319F1 | cactaggcatagaaaacagcaggaaaa |
| rs67861319F2 | ACGAGGCATAGAAAACAGCAGGAAAA |
| rs67861319R | GCTGGAGTGCAGTGATGCTACTATG |
| rs858678F | GAAAAGCCAGGATTGGGGTAGG |
| rs858678R | tcatgtggcacTGAACTATTCAGATAA |
| rs865750F | GAAGGAAAGGGCACCAGAAATTGTA |
| rs865750R | TAGTCTTTATGGGAGGGATGGCTGT |

F, forward; R, reverse.

Table S2. Ligation primer sequence

| Name | Sequence (5’-3’) |
| --- | --- |
| rs11570488FA | TGTTCGTGGGCCGGATTAGTttacacgtatgtgtcaccatgacA |
| rs11570488FG | TCTCTCGGGTCAATTCGTCCTTttacacgtatgtgtcaccatgacG |
| rs11570488FP | ggctaatttttgtaattttagtagagatagggtttTT |
| rs11570579FC | TTCCGCGTTCGGACTGATATAAGGATCTCAACCAAAGGTAACCAGAC |
| rs11570579FP | ATTCTTACACTCTGCTTAAGCAAACACTTAGAT |
| rs11570579FT | TACGGTTATTCGGGCTCCTGTAAGGATCTCAACCAAAGGTAACCAGAT |
| rs1713494RC | TTCCGCGTTCGGACTGATATCAAGCTTGGCCTATTTTATTTTTCCACG |
| rs1713494RP | TACCTACTTTCAAAGTCATTTAGGTATTTGAAGCTTTT |
| rs1713494RT | TACGGTTATTCGGGCTCCTGTCAAGCTTGGCCTATTTTATTTTTCCACA |
| rs221603RC | TCTCTCGGGTCAATTCGTCCTTGATTGTCTTCATTGTATTATTTCCTTGGTTTCATG |
| rs221603RP | TAGAGAAAAGTAATCAAGATCTAAGGAACTGACTT |
| rs221603RT | TGTTCGTGGGCCGGATTAGTGATTGTCTTCATTGTATTATTTCCTTGGTTTCGTA |
| rs67861319FA | TACGGTTATTCGGGCTCCTGTACAAATACATCCTATACGTTCAAAAACATAGAACAA |
| rs67861319FG | TTCCGCGTTCGGACTGATATACAAATACATCCTATACGTTCAAAAACATAGAACAG |
| rs67861319FP | Actggatgaggtggctcatgc |
| rs858678FC | TACGGTTATTCGGGCTCCTGTCTCTGAAGAAGGAGAAAAAAGAACTTCCATC |
| rs858678FG | TTCCGCGTTCGGACTGATATCTCTGAAGAAGGAGAAAAAAGAACTTCCATG |
| rs858678FP: | TTTACATATATGTATACATACACACACATACCTTTCTT |
| rs865750FA: | TGTTCGTGGGCCGGATTAGTtccgtctaaaacaaaaacaaaaacaaacaA |
| rs865750FC | TCTCTCGGGTCAATTCGTCCTTtccgtctaaaacaaaaacaaaaacaaacaC |
| rs865750FP | aaaactctataaaaagacaactgactgtttaaggcTTTTTTTTT |
